# *Leishmania* flagellum attachment zone is critical for flagellar pocket shape, development in the sand fly, and pathogenicity in the host

**DOI:** 10.1073/pnas.1812462116

**Published:** 2019-03-08

**Authors:** Jack D. Sunter, Ryuji Yanase, Ziyin Wang, Carolina Moura Costa Catta-Preta, Flavia Moreira-Leite, Jitka Myskova, Katerina Pruzinova, Petr Volf, Jeremy C. Mottram, Keith Gull

**Affiliations:** ^a^Department of Biological and Medical Sciences, Oxford Brookes University, Oxford OX3 0BP, United Kingdom;; ^b^Sir William Dunn School of Pathology, University of Oxford, Oxford OX1 3RE, United Kingdom;; ^c^Picobiology Institute, University of Hyogo, Hyogo 678-1297, Japan;; ^d^Centre for Immunology and Infection, University of York, York YO10 5DD, United Kingdom;; ^e^Department of Parasitology, Charles University, CZ-12844 Prague, Czech Republic

**Keywords:** *Leishmania*, pathogenicity, flagellar pocket, morphogenesis

## Abstract

*Leishmania* kinetoplastid parasites infect millions of people worldwide and have a distinct cellular architecture depending on location in the host or vector and specific pathogenicity functions. An invagination of the cell body membrane at the base of the flagellum, the flagellar pocket (FP), is an iconic kinetoplastid feature, and is central to processes that are critical for *Leishmania* pathogenicity. The *Leishmania* FP has a bulbous region posterior to the FP collar and a distal neck region where the FP membrane surrounds the flagellum more closely. The flagellum is attached to one side of the FP neck by the short flagellum attachment zone (FAZ). We addressed whether targeting the FAZ affects FP shape and its function as a platform for host–parasite interactions. Deletion of the FAZ protein, FAZ5, clearly altered FP architecture and had a modest effect in endocytosis but did not compromise cell proliferation in culture. However, FAZ5 deletion had a dramatic impact in vivo: Mutants were unable to develop late-stage infections in sand flies, and parasite burdens in mice were reduced by >97%. Our work demonstrates the importance of the FAZ for FP function and architecture. Moreover, we show that deletion of a single FAZ protein can have a large impact on parasite development and pathogenicity.

## Significance

*Leishmania* alternates between an insect vector and human host; in these different environments, the parasite adopts different forms. There are important commonalities between these different forms, particularly the flagellar pocket (FP) and associated flagellum attachment zone (FAZ). We show that the FAZ is important in different forms of *Leishmania* for FP shape and function, which are altered in mutants lacking a FAZ protein, FAZ5. FAZ5 deletion did not affect parasite proliferation and differentiation in culture; however, it dramatically reduced parasite proliferation in the sand fly and mouse. These results demonstrate the importance of the FAZ for FP function and architecture, and show that deletion of one FAZ protein can have a dramatic effect on *Leishmania* development and pathogenicity.

The eukaryotic parasites *Leishmania* are a group of species that infect millions of people worldwide and cause leishmaniasis, with symptoms ranging from cutaneous lesions to visceral infections (1). *Leishmania* species have a complex life cycle, adopting different shapes and forms as they alternate between an insect vector and a mammalian host (2). Within the sand fly vector, *Leishmania* is an extracellular parasite with a promastigote morphology characterized by an elongated body and a long motile flagellum. In contrast, within the mammalian host, *Leishmania* is an intracellular parasite that infects the macrophage and adopts an amastigote morphology, with a small rounded cell body and a flagellum that barely extends beyond the cell body. In both the promastigote and amastigote forms, there is an invagination of the plasma membrane at the base of the flagellum called the flagellar pocket (FP) (3). The FP is considered a key feature of the trypanosomatid cell and is central to processes that include endo/exocytosis, flagellum assembly, and the definition of surface membrane boundaries (4–6), which are critical for the cell biology underpinning the *Leishmania* life cycle.

The *Leishmania* FP has two distinct regions, a bulbous lumen that is ∼1 μm in length posterior to the FP collar (i.e., between the base of the flagellum and the collar) and a neck region where the FP membrane surrounds the flagellum more closely for a distance of ∼1 μm anterior to the FP collar, before the flagellum exits the cell body (3). The flagellum is attached to one side of the FP neck by the flagellum attachment zone (FAZ), which is a complex structure that connects the cell body cytoskeleton to the flagellum cytoskeleton, through the FP neck membrane and the flagellum membrane (3). The attachment of the flagellum to the FP neck creates asymmetry in the cell, with cytoplasmic structures organized in a defined pattern around the FP (3).

The FP is described as a key cellular feature enabling host–parasite interactions, but what is the evidence for this? There are only a few studies, and these address specific functions, such as the hemoglobin receptor, which localizes to the FP (7), yet the function of the overall cell biological organization of the FP has not been examined. Other studies of FP function in *Leishmania* have also focused on single proteins, such as ecotin-like serine peptidase inhibitor (ISP1) (8). Deletion of ISP1 altered the morphology of the anterior end of the cell body and resulted in the release of membranous material into the lumen. In the related species *Trypanosoma brucei*, research has focused on specific protein functions, such as BILBO1 and clathrin (9, 10). Knockdowns of either of these proteins are rapidly lethal in vitro, as they cause catastrophic changes in the overall FP architecture, and hence cannot be used to analyze FP function in vivo.

We have previously identified a series of *Leishmania* FAZ proteins that localize to the FAZ in the FP neck (3). Here, we show that deletion of one of these proteins, FAZ5, perturbed FP shape yet only had a small effect on endocytosis. The FAZ5 deletion mutant, while able to grow in culture, was unable to establish late-stage infections and develop in the sand fly, and showed a dramatic reduction in pathogenicity in the mouse.

## Results

### FAZ5 Null Mutants Have an Altered FP Shape and Size.

In *Leishmania*, the FAZ is present within the FP neck, and we hypothesized that the FAZ would have an important role in defining and maintaining FP shape. We targeted FAZ5 due to its localization along the entire length of the cytoplasmic side of the FAZ in the pocket neck (3, 11). We constructed viable FAZ5 null mutant promastigotes by replacement of the FAZ5 ORFs with antibiotic resistance markers in a parental cell line that expresses the flagellum membrane marker SMP1 tagged at its C terminus (with EGFP) and at the endogenous locus (12). SMP1 is a flagellum membrane protein, and the tagged version gives a readout of the organization of the FP region.

**Fig. 1.**
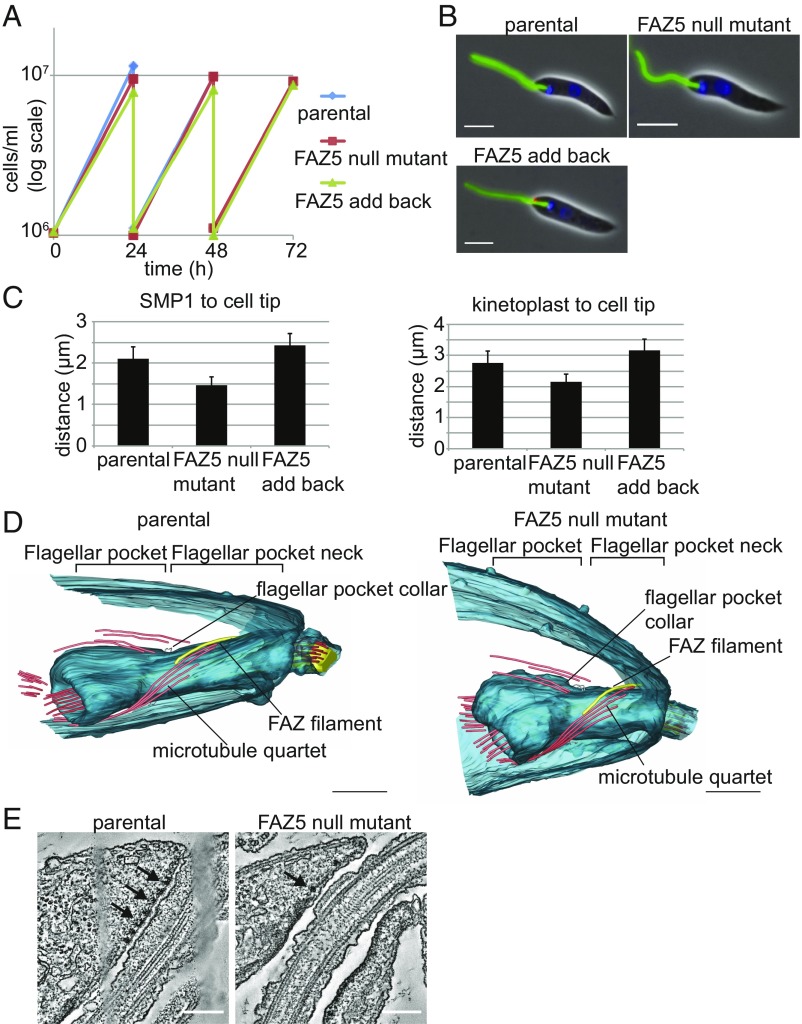
FAZ5 null mutant is able to grow but has a reduced length of flagellum within the cell body and a shorter FP. (*A*) Growth curves of the parental, FAZ5 null mutant, and FAZ5 add-back cells over a 72-h time period. (*B*) Light micrographs of parental, FAZ5 null mutant, and FAZ5 add-back cells labeled with SMP1::EGFP (green), FAZ5::mChFP add-back (red), and DNA (blue). (Scale bars, 5 μm.) (*C*) Measurement of flagellum length within the cell body as defined by the SMP1 signal and of the distance between the kinetoplast and the anterior cell tip for the parental, FAZ5 null mutant, and FAZ5 add-back cells. Fifty 1K1N cells were measured for each cell line, and the mean is plotted ± SD. (*D*) Models of FPs generated from tomograms of parental and FAZ5 KO cells. Note asymmetry of the flagellum exit point in the parental cell tomogram, which is lost in the FAZ5 null mutant tomogram. (Scale bars, 500 nm.) (*E*) Representative slices from the tomograms through the region of the FP neck, where flagellum attachment occurs. Electron-dense junctions connecting the membranes are clearly seen in the parental cells (arrows); however, in the FAZ5 KO cells (arrow), only a few electron-dense complexes are present, which are not associated with membrane connections. (Scale bars, 200 nm.) (Also refer to *SI Appendix*, Fig. S1.)

FAZ5 deletion was confirmed by PCR to check the integration of the resistance markers and loss of the ORF (*SI Appendix*, Fig. S1 *A* and *B*). Importantly, FAZ5 deletion had no effect on the growth rate of the cells in culture (Fig. 1*A*). The overall organization and morphology of the FAZ5 null mutant appeared normal by light microscopy (Fig. 1*B*); however, the SMP1 signal within the FP was shorter, with a reduction in the distance between the kinetoplast and the anterior cell tip, which corresponds to FP length (Fig. 1*C*). To confirm that this phenotype was specific to FAZ5 loss, we generated an add-back cell line with FAZ5 tagged at the C terminus with mChFP, using a constitutive expression plasmid (3). In the add-back cells, the distance between the proximal end of the SMP1 signal and the anterior cell tip was similar to that of the parental cells, indicating that the “shortening” of the FP was a specific effect of FAZ5 deletion.

We analyzed the morphology of the FP at the ultrastructural level using thin-section transmission electron microscopy (TEM) (*SI Appendix*, Fig. S1*C*). Longitudinal sections revealed that the overall FP layout was maintained, with both the neck region and the bulbous lumen present in the promastigote FAZ5 null mutant; however, both the distance between the basal body and the FP collar and that between the FP collar and the anterior end of the cell were smaller than in the parental cells (*SI Appendix*, Fig. S1 *C* and *D*). Transverse sections across the FP neck region showed that there was a loss of attachment between the flagellum and the FP neck in the FAZ5 null mutant (*SI Appendix*, Fig. S1*E*).

We used electron tomography to develop an overall 3D architecture of the FP (Fig. 1*D*). Tomograms of the mutant promastigotes confirmed that the overall organization of the pocket was unaltered in these cells; however, the FP length was reduced in the FAZ5 null mutant, with no attachment of the flagellum to the FP neck. Parental cells exhibit an asymmetrical end to the anterior cell body, with one side extending further along the flagellum than the other, resulting in the end of the cell body crossing the flagellum at an oblique angle when viewed in certain orientations. This asymmetry is missing in the FAZ5 null mutant (Fig. 1*D*). In parental cells, flagellum attachment to the cell body is mediated by a series of junctional complexes (Fig. 1*E*). In the FAZ5 null mutant, these electron dense junctional complexes were present and found close to the FP neck membrane; however, they were less numerous and did not appear to mediate attachment (Fig. 1*E*). The tomograms enabled us to examine FP cytoskeletal structures, such as the FP collar, microtubule quartet (MtQ), and FAZ filament (Fig. 1*D*). The FP collar in the FAZ5 null mutant was similar to that of parental cells, consisting of two filaments. The MtQ was still assembled in the mutant and followed the expected path around the bulbous lumen and up into the neck region. The FAZ filament was present in the FAZ5 null mutant, with its structure similar to that of the parental cells.

The TEM analysis gave a detailed description of the FP architecture changes in the FAZ5 null mutant. To provide molecular detail of the changes in the FP, we examined the TrypTag dataset and identified two potential markers of the FP (13). We endogenously tagged the *Leishmania* orthologs of these proteins (LmxM.23.0630–Sec10 and LmxM.06.0030) with a C-terminal mChFP tag in both the parental and FAZ5 null mutant cells (*SI Appendix*, Fig. S2*A*). LmxM.23.0630 localized to the bulbous domain of the FP in both the parental and FAZ5 null mutant cells. LmxM.06.0030 localized in the cytoplasm, with an enrichment at the FP neck region in the parental cells, yet the FP neck region signal was lost in the null mutant, providing further evidence for disruption of the FP architecture and organization in the FAZ5 null mutant.

### FAZ5 Deletion Affects the Overall FAZ Organization.

TEM revealed that flagellum attachment had been lost in the FAZ5 null mutant (Fig. 1*E*). The FAZ has multiple domains (14), with FAZ5 being a component of the cell body FAZ membrane domain. To examine the effect of FAZ5 deletion on other FAZ components, a series of FAZ proteins representing different FAZ domains were endogenously tagged with mChFP and their localization was examined (*SI Appendix*, Fig. S2*B*). The proteins tagged were FAZ1 and FAZ2, representing the cell body domain; ClpGM6 and FLA1BP, representing the flagellum domain; and FAZ10, which localizes to the anterior end of the cell at the flagellum exit point. FAZ1, FAZ2, ClpGM6, and FAZ10 are large proteins that contain predicted coiled-coil domains and have a structural function, whereas FLA1BP has predicted transmembrane domains and is important for forming the intramembrane connections (14–18). For the cell body domain FAZ proteins (FAZ1 and FAZ2), FAZ5 deletion caused the localization to change from a structured signal (a short line parallel to the flagellum for FAZ2 and a short line and ring across the flagellum for FAZ1) to a more disorganized and amorphous signal near the proximal end of the SMP1 signal (*SI Appendix*, Fig. S2*B*). This change in localization correlates well with the shortening of the FP neck and disorganization of the FAZ observed by TEM. For the flagellum domain FAZ proteins (ClpGM6 and FLA1BP), FAZ5 loss led to mislocalization of the proteins from a short line within the flagellum to either a cytoplasmic signal (for ClpGM6) or a signal found on the cell body membrane and flagellum membrane, with a concentration on the FP membrane (for FLA1BP) (*SI Appendix*, Fig. S2*B*). The FAZ10 localization did not change in the FAZ5 null mutant compared with the parental cell line.

### Perturbing FAZ Organization Affects Flagellum Length and Motility.

**Fig. 2.**
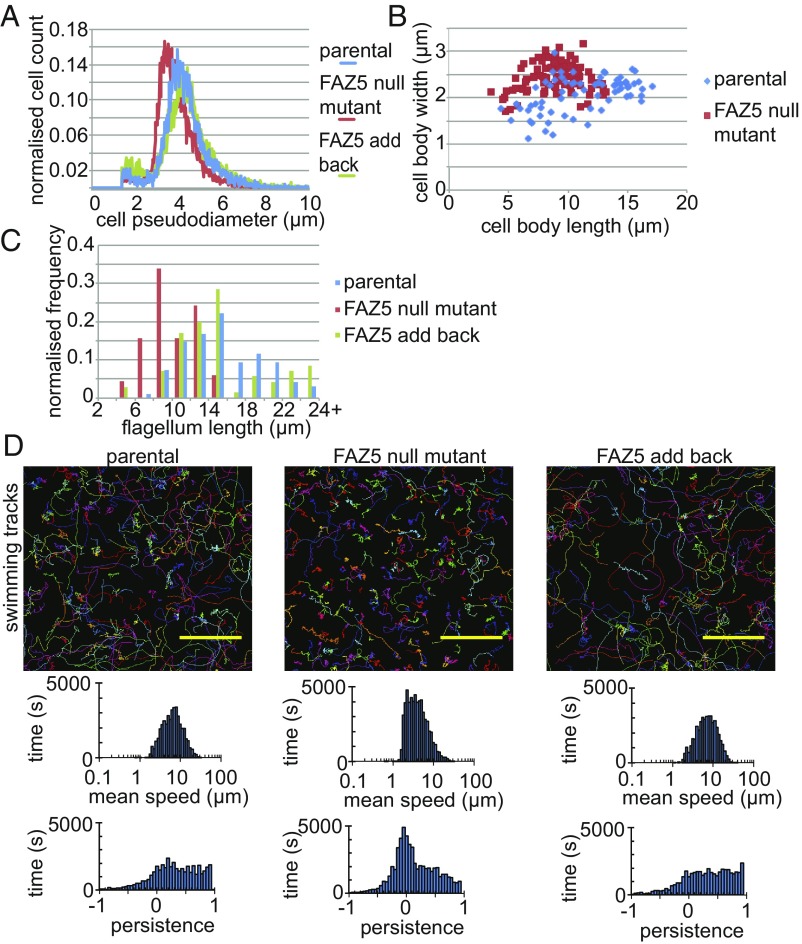
Deletion of FAZ5 changes the morphology and swimming of *Leishmania* cells. (*A*) Histogram of the pseudodiameter (a measure of cell volume) of the parental, FAZ5 null mutant, and FAZ5 add-back cells measured using a CASY cell counter. (*B*) Correlation of the cell body length and width of the parental, FAZ5 null mutant, and FAZ5 add-back cells. Fifty 1K1N cells were measured for each cell type. (*C*) Histogram of flagellum lengths of 1K1N cells for parental (*n* = 95), FAZ5 null mutant (*n* = 115), and FAZ5 add-back (*n* = 72) cells. (*D*) Swimming tracks from video microscopy of parental, FAZ5 null mutant, and FAZ5 add-back cells (*Upper*), with histograms of the mean speed and directional persistence for each cell line shown (*Lower*). (Scale bars, 50 μm.) (Also refer to *SI Appendix*, Figs. S2 and S3.)

Since the FAZ5 null mutant grew in vitro but had a changed FP shape, we asked whether there were any other cellular changes. The FAZ5 null mutant had a smaller volume than both the parental and FAZ5 add-back cells and was shorter and wider than the parental cells (Fig. 2 *A* and *B*). The mean flagellum length of the FAZ5 null mutant was significantly shorter than that of the parental cells (10.3 ± 2.4 μm vs. 15.4 ± 4.4 μm for FAZ5 null mutant and parental cells, respectively; *P* = 2.6 × 10^−21^, *t* test) (Fig. 2*C*). Moreover, no FAZ5 null mutants had flagella longer than 15 μm, whereas the parental and FAZ5 add-back cells were able to assemble flagella over 24 μm long.

Given that the FAZ5 null mutant had a defect in flagellum length control, we next examined its motility by tracking the movement of thousands of cells to calculate the mean speed and the directional persistence of the cells (19) (Fig. 2*D*). The FAZ5 null mutant had a mean speed similar to that of parental cells, but little processive movement. To examine this defect further, we analyzed the flagellar beat patterns of individual cells using a high-speed camera (*SI Appendix*, Fig. S3*A*). In parental cells, the majority of flagella had a regular flagellar beat, with some pausing and ciliary beats; however, in the FAZ5 null mutant, the majority of flagella beat in an uncoordinated manner. This lack of beat coordination is the likely explanation for the inability of the FAZ5 null mutant to swim processively. Importantly, the axoneme and paraflagellar rod (PFR) ultrastructure of the mutant and parental flagella were indistinguishable by TEM (*SI Appendix*, Fig. S3 *B* and *C*).

### FAZ5 Null Mutants Have a Reduced Rate of Endocytosis.

The FP is the sole site for exocytosis and endocytosis in *Leishmania*, and disruption of the FP architecture could affect both of these processes. To examine whether there was a defect in the transport of surface virulence markers, we examined the localization of lipophosphoglycan (LPG), gp63, and amastins (*SI Appendix*, Fig. S4). Monoclonal antibodies to LPG and gp63 were used to stain parental and FAZ5 null mutant cells. The localization and distribution of LPG and gp63 were unchanged (*SI Appendix*, Fig. S4*A*). We tagged three amastins (LmxM.29.0850, LmxM.08.0740, and LmxM.24.1270) representing the β-, δ-, and γ-amastin families (20) at their endogenous loci with a C-terminal dTomFP tag and examined their localization in promastigotes and axenic amastigotes (*SI Appendix*, Fig. S4*B*). For amastin 0850, the localization was similar between parental and mutant cells. In the promastigotes, the amastin was found on the cell body membrane and in the lysosome, and in the amastigote, amastin 0850 was found on the cell body membrane with a concentration in the cytoplasm near the posterior. The localization of amastin 0740 was similar in both the parental and FAZ5 null mutant promastigotes, with the amastin found on the cell body membrane and internal structures. In amastigotes, amastin 0740 was predominantly restricted to internal structures in both parental and mutant cells. For amastin 1270, there was a subtle difference in localization with a stronger signal on the cell surface and flagellum in the promastigote of the FAZ5 null mutant. In amastigotes, however, there was no observable difference, with amastin 1270 restricted to the lysosome.

**Fig. 3.**
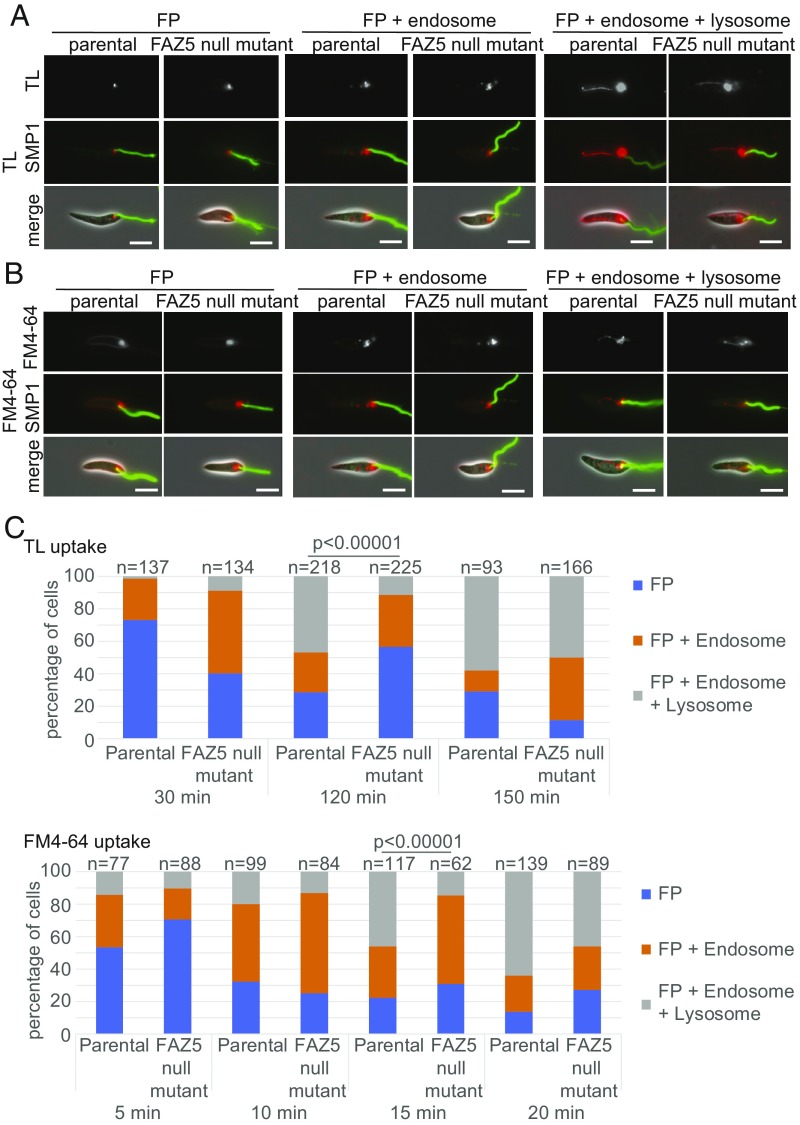
FAZ5 deletion reduces endocytosis rate. (*A* and *B*) TL and FM4-64 uptake assay with parental and FAZ5 null mutant promastigotes. Promastigotes were incubated with TL and FM4-64 over a 150-min time course and 20-min time course, respectively. Three major categories of TL and FM4-64 localization in the endocytic system were observed: FP; FP and endosome; and FP, endosome, and lysosome. The flagellum membrane protein SMP1 is tagged with EGFP (green). (Scale bars, 5 μm.) (*C*) Proportion of each category observed at each time point for both parental and FAZ5 null mutant cells for TL and FM4-64 uptake assays. Numbers counted for each time point are indicated above the columns. The uptake assays were done independently three times, and results from a representative experiment are shown. The *P* value was calculated using a χ^2^ test. (Also refer to *SI Appendix*, Figs. S4 and S5.)

To examine endocytosis in the parental and FAZ5 null mutant cells, we incubated them with fluorescently labeled dextran to monitor bulk fluid uptake, tomato lectin (TL) to monitor glycoprotein uptake, and FM4-64 to monitor plasma membrane uptake during endocytosis. All three markers took a consistent route through the endocytic system in both the parental and FAZ5 null mutant cells, although FM4-64 also tended to have a cell membrane signal (Fig. 3 and *SI Appendix*, Fig. S5). Initially, the marker was located within the FP at the base of the SMP1 signal. Next, the marker was observed in the endosomal system, a series of vesicular structures. Finally, the marker reached the terminal compartment of the endocytic pathway, which is the tubular lysosomal structure (21) that runs along the anterior/posterior axis of the cell. To quantify the rate of endocytosis, we examined the cells at multiple time points after the addition of the different markers and categorized the cells dependent on the signal observed into one of three categories: (*i*) FP signal only; (*ii*) FP and endosome signal; and (*iii*) FP, endosome, and lysosome signal (Fig. 3 and *SI Appendix*, Fig. S5).

Uptake of dextran was slow, with extended incubation times required for this marker to be seen in the lysosome (*SI Appendix*, Fig. S5*B*). However, when we compared the uptake in FAZ5 null mutant and parental cells, we observed that slightly fewer mutant cells had a lysosomal signal than the parental cells. Given the slow uptake of dextran, we concentrated on FM4-64 and TL, as we found they gave greater temporal resolution (Fig. 3). For both FM4-64 and TL, there was also a delay in the appearance of the marker in the lysosome of FAZ5 mutant cells in comparison to parental cells (Fig. 3 *B* and *C*). Moreover, the signal from the FP/endosome in the FAZ5 null mutant at the 120-min time point for TL and at the 15-min time point for FM4-64 is stronger than in the parental cells (*SI Appendix*, Fig. S5*C*). This suggests there is a delay in the transfer to the lysosome in the FAZ5 null mutant; however, the difference in endocytosis rate is small, as there was little difference in the distribution of the markers between the parental and FAZ5 null mutant cells by the end of the time course. To confirm the identity of the structures to which the endocytic markers localized, we endogenously tagged clathrin heavy chain at its C terminus with dTomFP as a marker for the endosome. We used the tagged amastin 1270 protein as a marker for the lysosome. Green fluorescent TL was incubated with these cell lines, and its localization was examined by fluorescence microscopy (*SI Appendix*, Fig. S5*D*). At early time points, there was overlap of TL with the clathrin signal, and at later time points, there was overlap of TL with the lysosomal amastin signal.

### FAZ5 Null Mutant Is Unable to Develop in the Sand Fly.

**Fig. 4.**
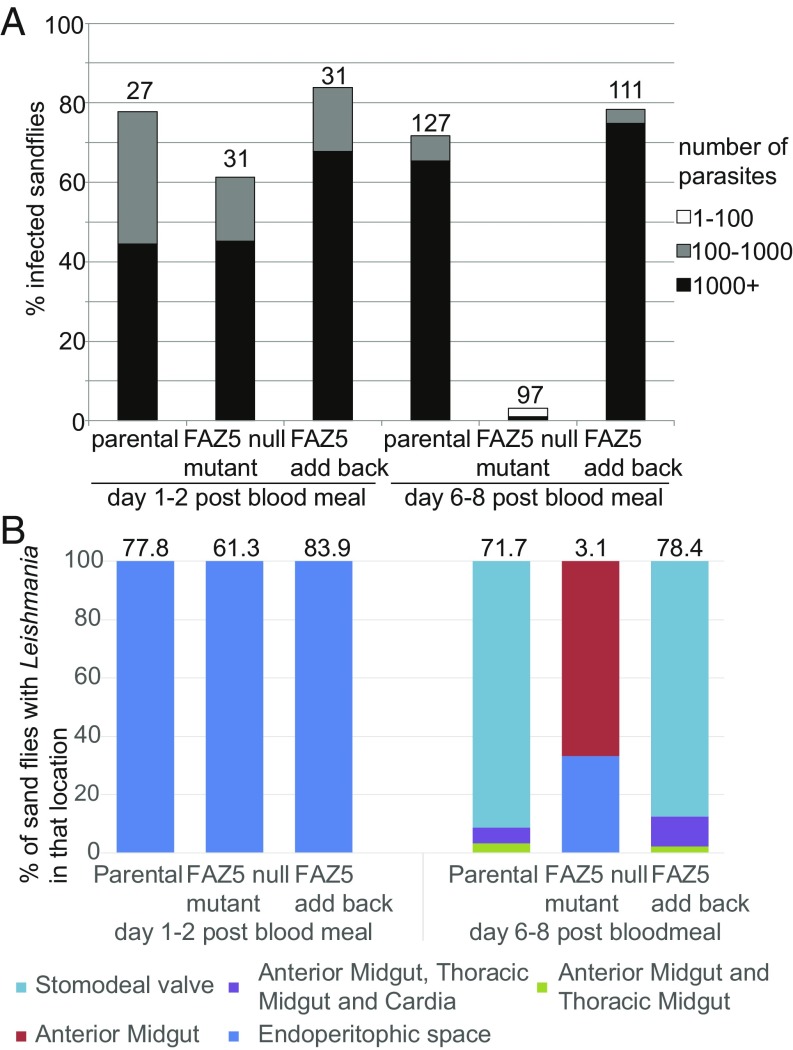
FAZ5 deletion severely affects the ability to proliferate and develop in the sand fly. (*A*) Analysis of sand fly infections using parental, FAZ5 null mutant, and FAZ5 add-back cells. At 1–2 d after a blood meal and 6–8 d after a blood meal, sand flies were dissected (numbers indicated above the columns) and the parasite load was measured as heavy (1,000+ parasites), moderate (100–1,000 parasites), or weak (1–100 parasites). (*B*) Location of *Leishmania* parasites within infected sand flies at 1–2 d and 6–8 d after a blood meal. Stacked columns indicate the percentage of infected sand flies, with parasites in various locations within the sand fly. The FAZ5 null mutant was unable to migrate to the stomodeal valve. The percentage of infected flies for each cell line is indicated above each column.

Despite the changes in FP architecture, FAZ5 deletion had no effect on promastigote growth in culture (Fig. 1*A*). However, this is a nutrient-rich, in vitro environment; thus, we wanted to ask if these promastigotes were able to thrive in the normal, more complex in vivo environments found in the vector where they attach to the midgut with their flagella. Hence, we infected sand fly vectors with the parental, FAZ5 null mutant, and FAZ5 add-back cells by feeding flies with blood containing these parasites. The sand flies were then dissected 1–2 d and 6–8 d after the blood meal, and the *Leishmania* parasite burden and location of parasites within the sand fly were assessed by light microscopy (Fig. 4 *A* and *B*). Two days after the blood meal, all of the cell lines were found in the midgut of the sand fly, proliferating within the blood meal. However, 8 d after the blood meal, the FAZ5 null mutant was practically undetectable in the sand fly midgut, whereas the parental and FAZ5 add-back cells were abundant throughout the sand fly midgut (Fig. 4*B*). Thus, the FAZ5 null mutant did not establish late-stage infections and had not migrated to the stomodeal valve, demonstrating that FAZ5 is critical for the full development program of *Leishmania* in its sand fly vector.

### FAZ5 Deletion Changes the Amastigote FP Shape and Dramatically Reduces Pathogenicity in Mice.

**Fig. 5.**
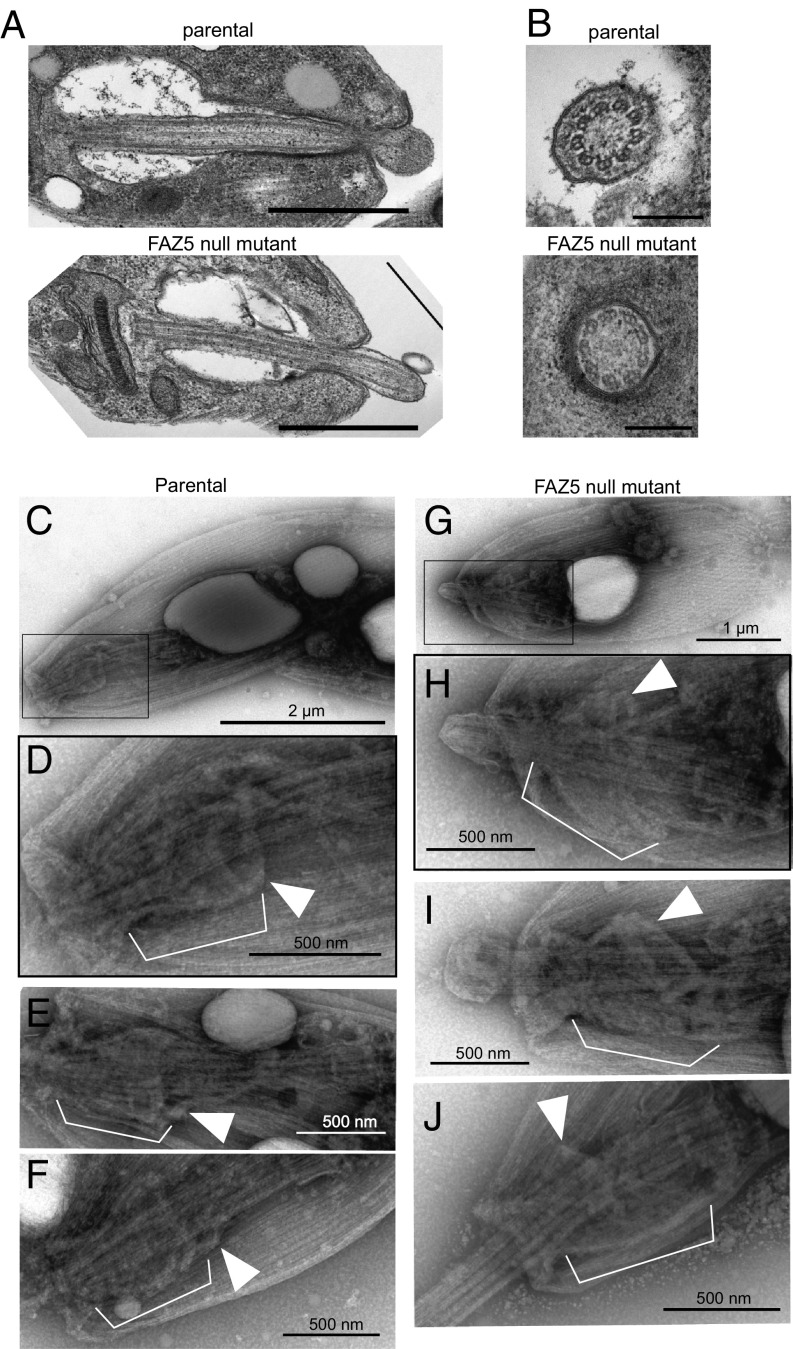
Deletion of FAZ5 dramatically alters the FP architecture in amastigotes. (*A*) Electron micrographs of longitudinal sections through the FP of parental and FAZ5 null mutant axenic amastigotes. (Scale bars, 500 nm.) (*B*) Electron micrographs of cross-sections through the axoneme, showing the lack of central pair microtubules. (Scale bars, 200 nm.) Whole-mount cytoskeletons of parental (*C*–*F*) and FAZ5 null mutant (*G*–*J*) axenic amastigotes were subjected to negative staining for observation by TEM. (*C* and *G*) Images of whole cells (with *Insets* in *D* and *H*, respectively) showing the position of the cytoskeletal structures displayed in higher magnification images. At the neck region of amastigote cytoskeletons, the axoneme is surrounded by a complex structure (white brackets). At its proximal end (relative to the flagellum base), this structure is delimited by filaments likely to correspond to the FP collar (white arrowheads). In parental cells, the neck cytoskeleton has a wine glass shape. In contrast, the neck cytoskeleton of FAZ5 null mutants appeared cup-shaped, with a considerably wider FP collar than that of parental cytoskeletons.

During differentiation from the promastigote form to the amastigote form, the FP is restructured, with the FP neck becoming more tightly apposed to the flagellum and the appearance of a constriction at the flagellum exit point (3, 22). In the amastigote, the flagellum has an enlarged tip and is much shorter, extending just beyond the cell body. The internal ultrastructure of the amastigote flagellum is radically different from that of the promastigote, as the axoneme does not have a central pair, the outer microtubule doublets are collapsed, and there is no PFR (22, 23). Differentiation to the amastigote form of the FAZ5 null mutant showed similar kinetics to those observed in the parental line. FAZ5 null mutant axenic amastigotes appeared similar by light microscopy to the parental cells; however, examination by TEM revealed that the FP of the FAZ5 mutant was dramatically different from that of the parental cells (Fig. 5*A*). The FP neck region was essentially missing, and the flagellum was no longer tightly apposed to the pocket membrane; instead, only the constriction at the distal end of the neck region was observed. This resulted in a larger bulbous lumen, which is different from that found in the promastigote phenotype (Fig. 5*A*; compare with Fig. 1*D*). Axonemes lacked the central pair as usual in amastigotes, and, interestingly, there was a longer region of flagellum beyond the cell body in the mutant (Fig. 5 *A* and *B*).

To investigate further the ultrastructural changes in the FP neck region, we examined detergent-extracted, negatively stained, whole-mount cytoskeletons (Fig. 5 *C*–*J*). The *T. brucei* flagellum, FAZ, and FP collar cytoskeleton can be visualized by negative-staining TEM after extraction with CaCl_2_, which depolymerizes the subpellicular microtubules (24). We attempted to use this approach to visualize the ultrastructure of the FAZ and collar cytoskeleton in amastigotes; however, the subpellicular microtubules of *Leishmania mexicana* were not sensitive to CaCl_2_. Despite the presence of the subpellicular microtubules, the observation of whole-mount cytoskeletons by negative-staining TEM revealed a distinctive structure at the amastigote neck region (Fig. 5 *C*–*F*). This structure is complex, consisting of multiple filaments, and is positioned around the anterior half of the axoneme, in the expected region of the FP neck. This structure, likely corresponding to the neck cytoskeleton, was delimited at its proximal end by filaments whose shape and orientation are similar to those of the FP collar. In addition, the diameter of this collar structure was 472 ± 70 nm in parental cells (*n* = 36), which is compatible with the width of the collar in parental cells by thin-section TEM (455 ± 83 nm). Careful comparison of the neck and collar cytoskeleton between parental and FAZ5 null mutant amastigotes revealed a clear difference in the shape of both the neck and the collar (Fig. 5 *C*–*J*). While the neck cytoskeleton had a “wine glass” shape in parental cells, becoming slightly narrower at the flagellum constriction point, the neck cytoskeleton of the mutant appeared “cup-shaped,” with a considerably wider FP collar opening than that of parental cells [472 ± 70 nm (*n* = 36) vs. 670 ± 136 nm (*n* = 57); *P* < 0.001, *t* test]. FAZ5 deletion resulted in a drastic change in the morphology of the neck and collar cytoskeleton of amastigotes.

**Fig. 6.**
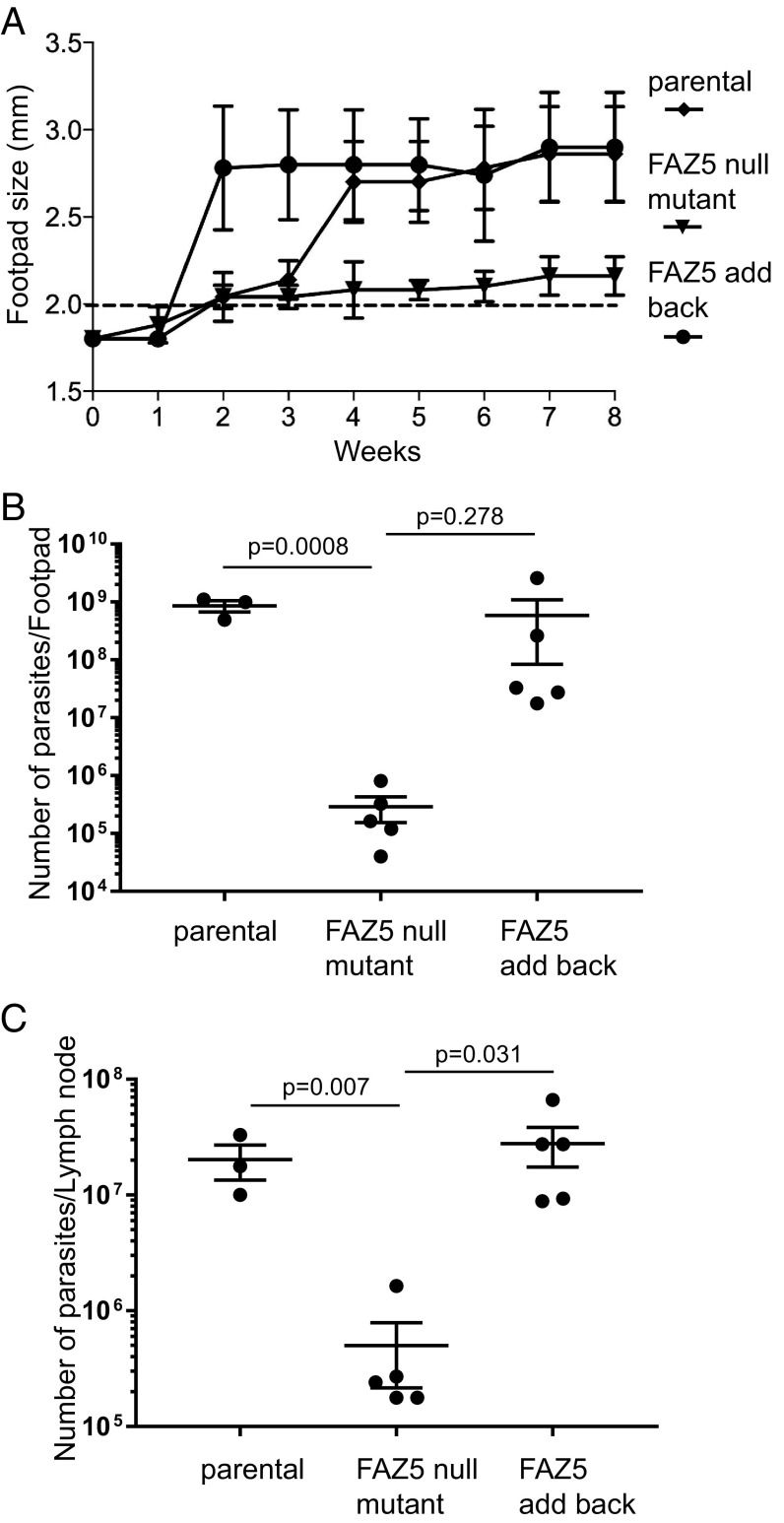
Deletion of FAZ5 causes a dramatic drop in virulence. (*A*) Measurement of mean footpad lesion size during an 8-wk infection time course with parental, FAZ5 null mutant, and FAZ5 add-back cells. Error bars represent SD. (*B* and *C*) Measurement of parasite burden at the end of the 8-wk infection time course in the footpad lesion and the lymph node for the parental, FAZ5 null mutant, and FAZ5 add-back cells. The parasite number from each infection is plotted, with the mean and the 95% SEM interval indicated. The *P* value was calculated using a two-tailed unpaired Student *t* test. (Also refer to *SI Appendix*, Figs. S6 and S7.)

To examine whether the FAZ5 null mutant had an altered ability to cause disease in a mammalian host, we performed footpad infections in a mouse model for cutaneous leishmaniasis (Fig. 6). Parental, FAZ5 null mutant, and FAZ5 add-back cells were injected into the footpad, and infection was monitored over an 8-wk time period by measuring the size of the footpad lesion (Fig. 6*A*). In comparison to the parental and FAZ5 add-back cells, the FAZ5 null mutant had much smaller lesions. At the end of the infection period, the parasite burden in the footpad and the lymph nodes was measured (Fig. 6 *B* and *C*). The mice infected with the FAZ5 null mutant had a >97% reduction of parasite numbers compared with those infected with the parental and FAZ5 add-back cells, which correlates well with the lesion size observed. Before *Leishmania* parasites are taken up by macrophages, they will be exposed to innate immune factors in the host. To investigate whether the FAZ5 null mutant was able to survive exposure to such factors, we incubated the parental, FAZ5 null mutant, and FAZ5 add-back promastigotes with media containing complete mouse serum over a 1-h time course (*SI Appendix*, Fig. S6*A*). All of the cell lines were susceptible to killing in the mouse serum; however, there were fewer FAZ5 null mutant cells alive after 1 h than was the case for the parental and FAZ5 add-back cells.

For a successful *Leishmania* infection to occur, the parasite has to enter a macrophage and proliferate within it. Therefore, an explanation for the lower parasitemia observed in the mouse infections could be that the FAZ5 null mutant does not infect macrophages. To assess the ability of these cells to infect macrophages, in vitro infections were performed with stationary-phase parental, FAZ5 null mutant, and FAZ5 add-back promastigotes (*SI Appendix*, Fig. S6 *B* and *C*). The FAZ5 null mutants were readily taken up by the macrophages, and the infection level at 24 h was higher than with the parental cells, potentially due to their defective motility. To examine whether there was a change in endocytosis rate in axenic amastigotes, we differentiated the parental and FAZ5 null mutant cells, and followed the uptake of FM4-64 (*SI Appendix*, Figs. S6 *D* and *E*). Over a 20-min time course, there was no apparent difference in FM4-64 uptake between the parental and FAZ5 null mutant amastigotes.

To confirm that the *Leishmania* promastigotes had differentiated to amastigotes and infected macrophages in the mouse, the infected footpads were dissected and processed for TEM (*SI Appendix*, Fig. S7). In the infections with parental cells, infected macrophages were readily observed and parasitophorous vacuole sections showed multiple parasites indicative of proliferation. The FAZ5 null mutant material had more lysed cells, and only a few parasites were seen despite extensive searching. Sections through the FP of the FAZ5 null mutant parasites revealed an organization similar to that of the parental cell, including an FP neck. However, the mutant cells appeared to have a larger bulbous FP lumen, with the mean distance across the widest section of the lumen twice as wide in the mutant than in the parental cells [1,125 ± 139 nm vs. 564 ± 68 nm in mutant and parental amastigotes, respectively, in vivo (*n* = 5–8 cells); *P* = 0.004, Mann–Whitney *U* test].

## Discussion

Here, we demonstrate the importance of the FAZ for FP architecture and function in *Leishmania*. FAZ proteins and structure were first studied in extensive detail in *T. brucei*, where the FAZ has a major role in attaching the near-full flagellum length to the cell body. In *T. brucei*, the disruption of almost any part of the FAZ structure produces gross changes and lethality (14). However, the discovery of the more restricted localization of the FAZ protein complexes in *Leishmania* (3) provided an opportunity for us to examine more discrete dependency relationships in cell morphogenesis, but also the larger question of how these changes affect proliferation and development in the mammalian host and insect vector. Deletion of FAZ5 in *Leishmania* produced a clear defect in FP architecture, with minor consequences for endocytosis; however, this mutant was fully competent for both proliferation and differentiation from promastigote to amastigote in culture. Importantly, disruption of a single FAZ protein dramatically reduced pathogenicity in mice and parasite establishment in the vector. The effect of FAZ disruption on the FP and pathogenicity supports the notion that the FP is a key host–parasite interface.

### FAZ Has a Key Role in Cell and FP Morphogenesis.

In the FAZ5 null mutant, the FP length was shorter, resulting in a reduction of the portion of the flagellum found within the cell body, with the kinetoplast located closer to the anterior cell tip. The flagellum was also no longer attached to the cell body in the FP neck region; as FAZ5 has multiple predicted transmembrane domains, this suggests that FAZ5 has a key function in forming the connections between the cell body membrane and the flagellum membrane within the FAZ (3, 11). The FAZ5 null mutant provides insights into the hierarchy and dependencies of FAZ assembly, as the loss of FAZ5, a cell body membrane domain component of the FAZ, affects the localization of FAZ proteins in other FAZ domains differentially. The cell body FAZ domain proteins, FAZ1 and FAZ2, still localized to the FAZ, albeit with a smaller focus of signal; this correlates with electron tomography showing that the FAZ filament and MtQ were still assembled. Therefore, the assembly of the cell body FAZ domain is not dependent on the intramembrane FAZ domain. However, the flagellum FAZ domain proteins FLA1BP and ClpGM6 were not localized at the FAZ, which suggests that this FAZ domain is dependent on the assembly of the intramembrane FAZ domain. Deletion of FAZ5 also affected the localization of the FP neck marker LmxM.06.0030. However, there was no effect on the localization of LmxM.23.0630, a marker of the bulbous domain of the FP and a component of the exocyst complex, suggesting that large changes in exocytic activity were unlikely.

Loss of FAZ5 affects not only the structure and organization of the FP and associated FAZ but also the overall shape and size of the promastigotes. The FAZ5 null mutant was shorter and wider, with a smaller overall volume than the parental cells. Interestingly, in *T. brucei*, changes in FAZ length are also associated with changes in cell body length (17, 25, 26). This suggests that cell size control is a conserved function of the FAZ across the different trypanosomatid species, in addition to connecting the flagellum to the cell body.

The FAZ5 null mutant was unable to assemble a flagellum of the same length as that of parental cells, with the mean flagellum length being 5 μm shorter. Furthermore, the shorter flagella were generally not capable of beating in a coordinated manner, which meant that the FAZ5 null mutant was able to move, but not in a consistent direction. We presume that this aspect of the FAZ5 FP phenotype reflects the dependency of the flagellum FAZ domain, as well as flagellum assembly and function, on the link with the cell body via the intramembrane FAZ domain, which is affected by FAZ5 deletion. In addition, the physical connection itself between the flagellum and the cell body mediated by the FAZ may have a role in coordinating the flagellar beat, and the loss of this connection in the FAZ5 null mutant could contribute to the uncoordinated flagellar beat.

### Lack of Development in the Sand Fly Vector.

Despite the changes to the FP architecture, cell shape, and flagellum, there was no difference in proliferation in vitro between the FAZ5 null mutant and the parental cells. However, in vitro growth conditions do not replicate the myriad challenges presented by the life cycle of these parasites. This difference between in vitro culture and the in vivo environments is clearly highlighted by the FAZ5 null mutant, which was unable to develop within the sand fly. In a normal sand fly infection, the *Leishmania* parasites are taken up with the blood meal, which is then surrounded by a peritrophic matrix within the sand fly gut, with subsequent blood meals enhancing *Leishmania* transmission and infectivity (27). Initially, the parasites develop and proliferate within the blood meal; however, they must escape before the blood meal remnants are defecated (28, 29). This escape requires the parasites to move and attach to the microvilli of the midgut epithelium. This binding is stage-dependent, being limited to the forms found in the middle phase of development and facilitated by sand fly midgut mucin (30, 31). The FAZ5 null mutant proliferated within the blood meal (early-stage infection); however, very few parasites were observed once the blood meal had been defecated, suggesting that these cells were not able to escape and attach to the midgut microvilli to establish late-stage infections. The loss of directional movement in the FAZ5 null mutant would presumably compromise its ability to escape the blood meal and attach; thus, it would be cleared by defecation. There are likely to be additional factors related to the changes in FP shape caused by targeting of the FAZ that have an impact on the development of the parasite. The distribution of the surface-expressed virulence factors, gp63, LPG, and amastins was not affected in the FAZ5 null mutant. However, the endocytic rate of the FAZ5 null mutant was reduced, and while this did not affect growth in culture, it may have an impact on development in the sand fly by altering the uptake of nutrients or reducing the rate at which deleterious material, such as complement components in the blood meal, can be internalized and digested. The reduction in endocytic rate observed in the FAZ5 null mutant suggests a potential connection between FP architecture and one of its key functions. Furthermore, one might also conjecture that the exit point shape change from an asymmetrical to a symmetrical shape may have specific effects on the entry of substances.

### Dramatic Reduction of Pathogenicity in the Mammalian Host.

The FAZ5 null mutant had dramatically lower pathogenicity in the mouse, with smaller footpad lesions and a >97% reduction in parasite burden within both the footpad and the lymph nodes. This drop in pathogenicity is unlikely to be related to the motility problems observed in the null mutant, as the promastigotes were able to infect macrophages in vitro. In fact, the initial rates of infection were higher, as these parasites were unable to move away effectively from the macrophages. Furthermore, the FAZ5 null mutant was able to differentiate normally to amastigotes in vitro and in the mouse, as amastigotes were found in the infected footpads. However, the FAZ5 null mutant promastigotes were more susceptible to killing by mouse serum, but this effect was minor in comparison to the reduction in parasite burden between the parental and FAZ5 null mutant cells in the mouse. Given that the mutant was able to infect macrophages and differentiate into amastigotes, the drop in pathogenicity in the mouse is likely to be caused by a reduction in parasite proliferation mediated by the host and not by a defect in cell division.

The FAZ5 null mutant axenic amastigote FP had a radically different architecture from that of the parental cells. The diameter of the FP collar was substantially larger in the mutant, which resulted in the FP neck region being essentially missing; hence, the cells no longer have the FP membrane tightly apposed to the flagellum. This was reflected in the whole-mount cytoskeletons, where the cytoskeletal structures associated with the FP neck region were no longer closely positioned around the axoneme. We examined the overall FP structure of the few FAZ5 null mutant parasites present in the footpad infections. Interestingly, although these cells were rare, they seemed to have an FP neck region whose length and shape were more akin to those of the parental cells, but they had a larger FP bulbous lumen. Since these are deletion mutants, there is no question of a reversion genotype; however, it is, of course, possible that these rare cells have acquired this modified FP architecture via compensatory mutations or phenotypic plasticity during construction. However, since the FAZ5 null mutant had previously been passaged through mice, isolated, and transformed into promastigotes before being used for these experiments, the acquisition of this modified FP, even if it does enable faster proliferation, does not appear to be stably inherited. Interestingly, there is an increase in lesion size in the FAZ5 null mutant infection after week 6, which might indicate an adaptation enabling faster proliferation.

FAZ5 deletion caused a large change in FP architecture, which will likely have an effect on FP function. The FP is the site of the membrane domain boundaries that delineate the flagellum, FP, and cell body membranes (5, 6). The localization of the flagellum membrane protein SMP1 is not altered in the FAZ5 null mutant, suggesting that these boundaries are still intact. However, the FAZ5 null mutant has an altered flagellum membrane domain organization, as shown by the disrupted localization of FLA1BP, that could impact other proteins, with consequences for parasite proliferation and/or macrophage immunological response to the infection. Within the macrophage, the FP will have a vital role in sculpting the environment of the parasitophorous vacuole, creating a suitable environment for parasite proliferation (32, 33). This will require both the uptake and secretion of material via the FP; there was no change in uptake of FM4-64 in axenic amastigotes, suggesting that these processes have not been greatly disrupted. However, the loss of the tight FP neck region may reduce the ability of the parasite to control the FP contents, resulting in exposure to harmful material.

The FAZ is a specialized membrane–membrane junction, and such junctions also occur in multicellular organisms, where they are also mediated by complex cytoskeletal structures. Tight junctions are important for defining the apical and basolateral portions of epithelial cells by controlling the protein complement in each membrane region (34). Moreover, desmosomes, another type of membrane–membrane junction, are important for connecting cells together (35). Clearly, despite the evolutionary distance between the kinetoplastids and multicellular organisms, these junctional complexes have similar functions to perform, and insights here into defining membrane architecture could have implications in other eukaryotes.

Deletion of FAZ5 caused a perturbation in FP shape with a modest reduction in endocytosis rate, and this mutant is sustainable in culture, yet is unable to proliferate effectively within the sand fly vector or mammalian host. Thus, a FAZ mutation causing a discrete change in the FP shape affected the entire cycle of this human disease, demonstrating the importance of the FAZ for *Leishmania* pathogenicity.

## Materials and Methods

### Cell Culture.

*L. mexicana* (WHO strain MNYC/BZ/1962/M379) promastigotes were grown at 28 °C in M199 medium with Earle’s salts, l-glutamine, 10% FCS, 40 mM Hepes-NaOH (pH 7.4), 26 mM NaHCO_3_, and 5 μg/mL hemin. Cells were maintained in logarithmic growth by regular subculturing. Promastigotes were differentiated from axenic amastigotes by subculturing into Schneider’s *Drosophila* medium with 20% FCS and 25 mM MES⋅HCl (pH 5.5) at 34 °C with 5% CO_2_ and grown for 72 h without subculture.

### Generation of FAZ5 Deletion Constructs, Tagging Constructs, and FAZ5 Add-Back Construct.

Deletion constructs were generated using fusion PCR as described (36). Five hundred base pairs of the 5′ UTR and 500 bp of the 3′ UTR of the *FAZ5* gene were combined with either the hygromycin resistance gene or the bleomycin resistance gene by PCR to generate the deletion constructs. For tagging, the corresponding ORFs and UTRs were cloned into pLEnTv2-YB, pLEnTv2-mChP, or pLEnTv2-dTP plasmid (3, 36). The modular constitutive expression plasmid has been described previously (3), with the exception that the *eYFP* gene and following intergenic sequence were replaced with the *mChFP* gene and the *L. mexicana histone 2B* intergenic sequence in this study. The *FAZ5* gene was then cloned upstream using the HindIII and SpeI restriction sites. Constructs were electroporated using a Nucleofector 2b device (36).

### Light Microscopy.

For live cell microscopy, cells were washed three times in PBS and resuspended in PBS with Hoescht 33342 (1 μg/mL); 5 μL was then placed on a Polysine slide. The cells were imaged using either a Leica DM5500B microscope with a 100× objective and Neo 5.5 sCMOS camera or a Zeiss ImagerZ2 microscope with a 63× or 100× objective and Hamamatsu Flash 4 camera. For immunofluorescence, cells were harvested by centrifugation (800 × *g* for 5 min) and washed twice in PBS. Cells were settled onto a Polysine slide for 5 min, and 4% formaldehyde in PBS was added to the slide (final concentration of 2%) for 5 min before adding 1% glycine and 0.1% Nonidet P-40 for permeabilization. Slides were washed in PBS and blocked with 1% BSA in PBS for 30 min. A primary antibody against gp63 (Gene Tex) and LT22 (37) were added at a 1:1,000 ratio in 1% BSA for 1 h. Slides were washed in PBS before incubation with the secondary antibody, TRITC-conjugated anti-mouse IgG, at a 1:200 ratio in 1% BSA for 45 min. Slides were washed in PBS and mounted before imaging. Swimming and flagellar beat behaviors were analyzed for cells as described (19). For cell-swimming analysis, a 25.6-s video at five frames per second under dark-field illumination was captured using a 20× objective. Particle tracks were traced automatically, and mean cell speed, mean cell velocity, and cell directionality (the ratio of velocity to speed) were calculated. For flagellar beat analysis, a 4-s video at 200 frames per second under phase-contrast illumination was captured from a thin film of cell culture between a slide and coverslip using a Zeiss Observer microscope with a 20× objective and an Andor Neo 5.5 camera. Flagellar beats for each cell were classified manually.

### TEM.

Cells were fixed in culture by the addition of glutaraldehyde to give a final concentration of 2.5%. After 3 min, the cells were centrifuged (at 900 × *g* for 5 min), washed in buffered fixative solution [0.1 M piperazine-*N*,*N*′-bis(2-ethanesulfonic acid) (PIPES)–NaOH buffer (pH 7.2), with 2.5% glutaraldehyde and 4% formaldehyde], resuspended in fresh buffered fixative solution, and fixed overnight at 4 °C. Cells were then washed five times in 0.1 M PIPES-NaOH buffer (pH 7.2, including one 30-min wash in 50 mM glycine in 0.1 M PIPES-NaOH buffer), and postfixed in 1% OsO_4_ in 0.1 M NaOH buffer at 4 °C for 2 h. Cells were washed five times in deionized water and then stained *en bloc* with 2% aqueous uranyl acetate overnight at 4 °C. Samples were then dehydrated in ethanol and embedded in Agar 100 resin. Thin sections were stained with Reynolds’ lead citrate before imaging on a Tecnai T12 microscope equipped with a OneView 4 × 4 megapixel camera (Gatan).

Dissected tissues were fixed in primary fixative (2.5% glutaraldehyde and 4% paraformaldehyde in 100 mM cacodylate buffer) for 1 h. The tissues were washed once with 100 mM cacodylate buffer and transferred into the secondary fixative (1% osmium tetroxide in 100 mM cacodylate buffer) and incubated for 1 h. The tissues were washed twice with 100 mM cacodylate buffer and then stained *en bloc* with 1% uranyl acetate in 50% ethanol for 5 min. The stain was removed, and this step was repeated. Samples were then dehydrated in ethanol and embedded in resin. Thin sections were stained with Reynolds’ lead citrate before imaging.

For negative staining, axenic amastigotes were centrifuged at 800 × *g* for 5 min and resuspended in 0.5 mL of culture medium. Cells were allowed to adhere to formvar/carbon-coated and -charged nickel grids (0.8 μL of cell suspension per grid) for 2 min, and then treated sequentially with 1% IGEPAL in PEME [0.1 M PIPES (pH 6.9), 2 mM EGTA, 1 mM MgSO_4_, 0.1 mM EDTA] for 5 min and with 60 mM CaCl_2_ for 2 min before fixation in 2.5% glutaraldehyde in PEME for 10 min. Then, grids were washed once in ddH_2_O and stained with 1% aurothioglucose (USP; Merk) before observation in an FEI Tecnai T12 transmission electron microscope.

### Electron Microscopy Tomography.

Ribbons containing serial sections of ∼150 nm were produced from samples prepared for TEM. Sections were stained with Reynolds’ lead citrate before imaging at 120 kV on a Tecnai T12 microscope with a OneView Gatan camera. Each individual tomogram was produced from a total of 240 4 × 4 megapixel images (120 tilted images each of 0° and 90° axes, with 1° tilting between images) acquired automatically using SerialEM. Individual tomograms were produced using eTOMO (IMOD software package), and consecutive tomograms were then joined to produce serial tomogram volumes using eTOMO. Tridimensional models from serial tomograms were produced by manual tracing and segmentation of selected structures using 3Dmod (IMOD software package).

### Endocytosis Assays.

Promastigotes (5 × 10^6^ cells) were incubated in complete M199 medium with either FM4-64 (40 μM), 500 μg/mL 10-kDa Dextran-Texas Red, or 1 μL of Dylight 488- or Dylight 594-conjugated TL (1-mg/mL solution; Vector Labs) at 28 °C. At each time point, cells were removed and washed with PBS before imaging. Amastigotes (1 × 10^7^ cells) were incubated in Schneider’s medium with FM4-64 (40 μM) at 34 °C. At each time point, cells were removed and washed with PBS before imaging.

### Sand Fly Infections.

All parasites were cultivated at 23 °C in M199 medium supplemented with 20% FCS, 1% basal medium Eagle vitamins, 2% sterile urine, and 250 μg/mL amikin. Before infections, parasites were washed three times in saline and resuspended in defibrinated heat-inactivated rabbit blood at 10^6^ promastigotes per milliliter. *Lutzomyia longipalpis* was maintained at 26 °C and high humidity on 50% sucrose solution and for a 14-h light/10-h dark photoperiod. Sand fly females, 3–5 d old, were fed through a chick skin membrane (38). Fully engorged females were separated and maintained at 26 °C with free access to 50% sucrose solution. They were dissected on days 1–2 and 6–8 after a bloodmeal, and the guts were checked for localization and intensity of infection by light microscopy. Parasite loads were graded as described previously (39). Each cell line was used to infect sand flies in two independent experiments.

### Serum Killing.

A total of 5 × 10^6^ cells were collected via centrifugation and resuspended in 5 mL of Hanks’ buffered salt solution containing either 20% (vol/vol) mouse serum (Sigma–Aldrich) or 20% (vol/vol) heat-inactivated FCS (Gibco). The cells were incubated for 1 h at 37 °C; aliquots were taken after 20, 40, and 60 min; and live cells were counted using a hemocytometer.

### Macrophage Infections.

Bone marrow-derived macrophages (BMDMs) were grown in DMEM with 10% FCS and 10 ng/mL macrophage-CSF at 37 °C with 5% CO_2_. BMDMs were grown to confluence and then used to seed wells at 2.5 × 10^4^ cells per well. Promastigotes in log growth were split into 1 × 10^5^ cells per milliliter and grown to stationary phase over 5 d. The stationary-phase promastigotes were used to infect the BMDMs for 2 h at a multiplicity of infection of 5. After washing the cells to remove any free parasites, the infected BMDMs were incubated at 34 °C with 5% CO_2_ in DMEM for 3 d. At each time point, BMDMs were fixed with methanol, stained with DRAQ5, and then imaged. Infected BMDMs and *Leishmania* parasites were then counted.

### Virulence Assessment in Vivo: Footpad Measurement and Limiting Dilution Assays.

All experiments were conducted according to the Animals (Scientific Procedures) Act of 1986, United Kingdom, and had approval from the University of York Animal Welfare and Ethical Review Body (AWERB) committee. Strain virulence was assessed by footpad swelling and parasite burden (40). For experimental infections, the parasites had previously been passaged through mice, isolated, and transformed into promastigotes before being used. Groups of five female BALB/c mice (4–6 wk of age) were infected s.c. at the left footpad using 2.0 × 10^6^ stationary promastigotes in 40 μL of sterile PBS. Infections were followed weekly by footpad measurement, and animals were culled after 8 wk using approved schedule 1 methods before removal of footpad lesions and lymph nodes under sterile conditions. Samples were kept in M199 supplemented with 5 μg/mL gentamycin, and footpads were digested with 4 mg/mL collagenase D for 2 h at 37 °C. Lymph nodes and digested tissues were mechanically dissociated and filtered through a 70-μm cell strainer (BD Biosciences). Homogenates were resuspended in M199 supplemented with 20% FCS, and serial dilutions (twofold) were performed in 96-well, clear, flat-bottomed plates. Each sample dilution was performed in duplicate and distributed in at least three plates. Sealed plates were incubated for 7–10 d at 25 °C, wells were visually analyzed for the presence of parasites, and the number of parasites was calculated by multiplying by the dilution factors.

## Supplementary Material

Supplementary File
