# Supplementary material for: Leishmania flagellum attachment zone is critical for flagellar pocket shape, development in the sand fly, and pathogenicity in the host
Source: Proc Natl Acad Sci U S A. 2019 Mar 8;116(13):6351–60. doi: 10.1073/pnas.1812462116 (PMC6442623; doi:10.1073/pnas.1812462116)
Supplement: Supplementary File [file pnas.1812462116.sapp.pdf]

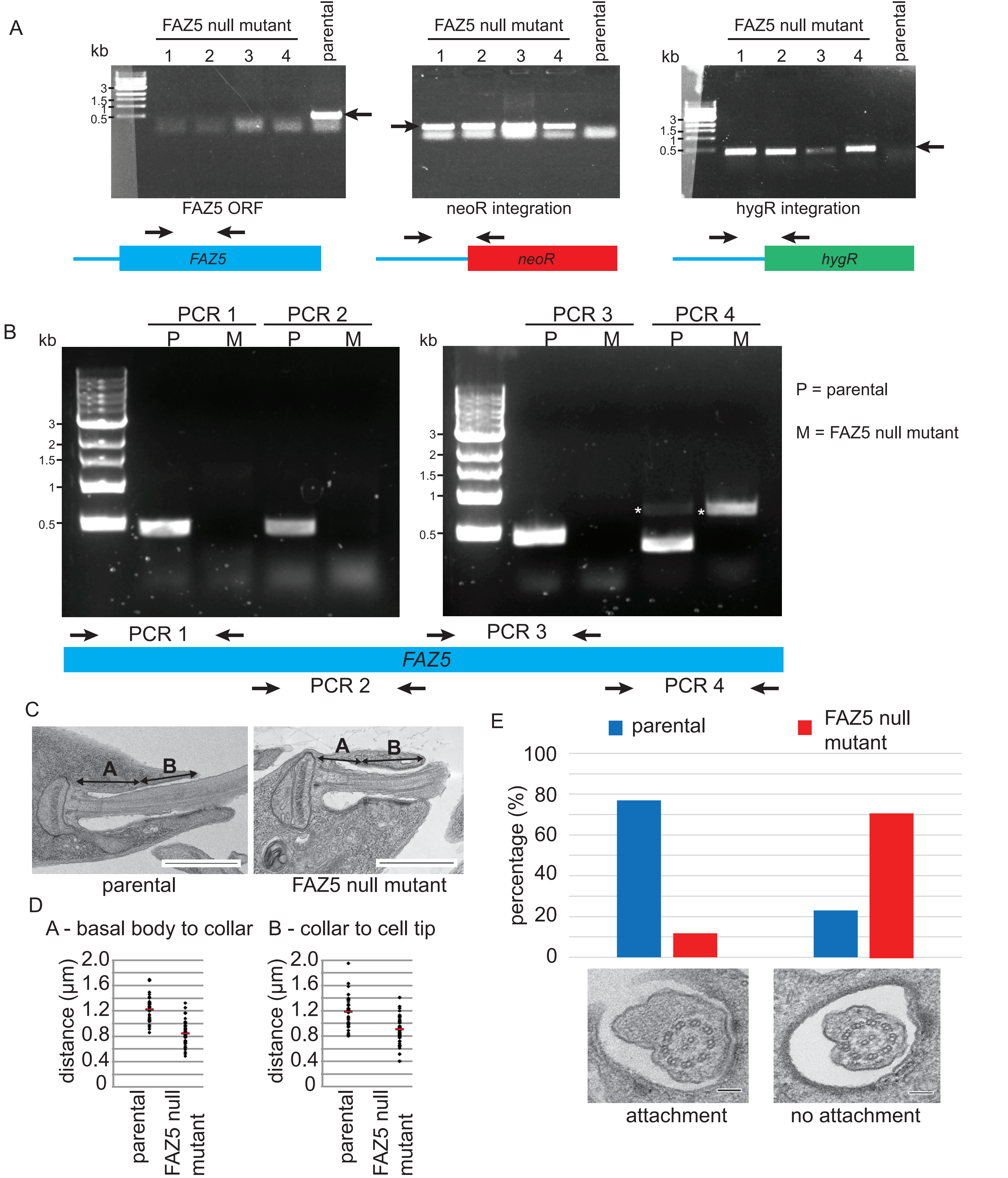

Figure S1 related to Figure 1. A) Confirmation of *FAZ5* gene deletion. gDNA from 4 null mutant clones and the parental cells was analysed by PCR. PCR confirmed that FAZ5 ORF was no longer present in the null mutant clones and that the resistance markers had integrated correctly. FAZ5 null mutant clone 1 was used for all subsequent experiments. B) PCR based scan across the FAZ5 ORF. The FAZ5 ORF was split into ~500 bp fragments and primers used to amplify each segment fromparental and FAZ5 null mutant clone 1 gDNA. In PCR 4 a non-specific band (white asterisk) was amplifed in addition to the expected band from parental gDNA and from the FAZ5 null mutant only the non-specific band was amplified. C) Representative electron micrographs of longitudinal section through the FP of a parental cell and FAZ5 null mutant. A represents the distance between the basal body and the FP collar and B represents the distance between the FP collar and the anterior cell tip. Scale bar is 1 µm. D) Measurement of A and B highlighted in (C). Each measurement (parental n=37, FAZ5 null mutant n=50) is plotted with the mean represented as a red line. E) Quantitation of the different transverse profiles across the flagellum and cell body observed as the flagellum extends through the FP into the FP neck region between the parental cells (n=39) and FAZ5 null mutant (n=34). Electron micrographs below illustrate the two profiles based on the presence or absence of attachment between the flagellum and the cell body. Scale bar is 100 nm.

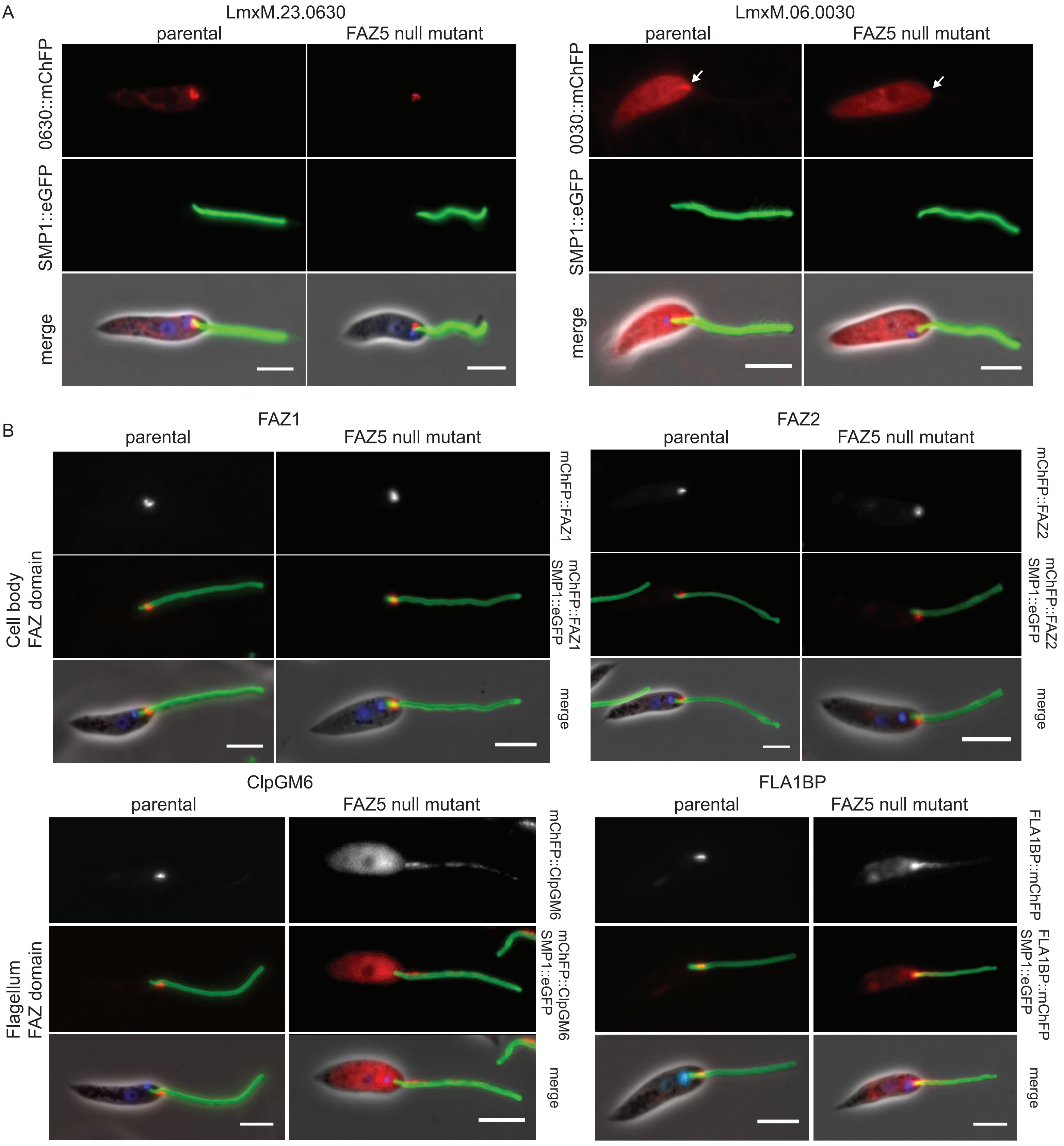

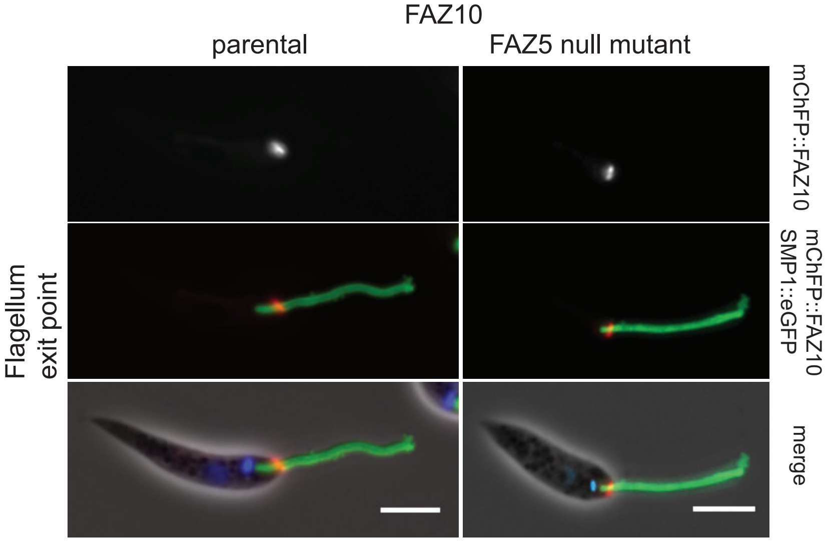

A) Figure S2 related to Figure 1.
Images of FP markers LmxM.23.0630 and LmxM.06.0030 tagged with mChFP (red) in parental and FAZ5 null mutant cells. LmxM.23.0630 marks the bulbous reigon of the FP. LmxM.06.0030 marks the FP neck region in the parental cells (white arrow) but is not present in the FAZ5 null mutant (white arrow). The flagellum membrane protein SMP1 is tagged with eGFP (green) and the DNA is stained with Hoescht 33342 (blue). Scale bar represents 5 µm. B) Images of FAZ proteins tagged with mChFP (red) representing the different FAZ domains in parental and FAZ5 null mutant cells. The flagellum membrane protein SMP1 is tagged with eGFP (green) and the DNA is stained with Hoescht 33342 (blue). Scale bar represents 5 µm.

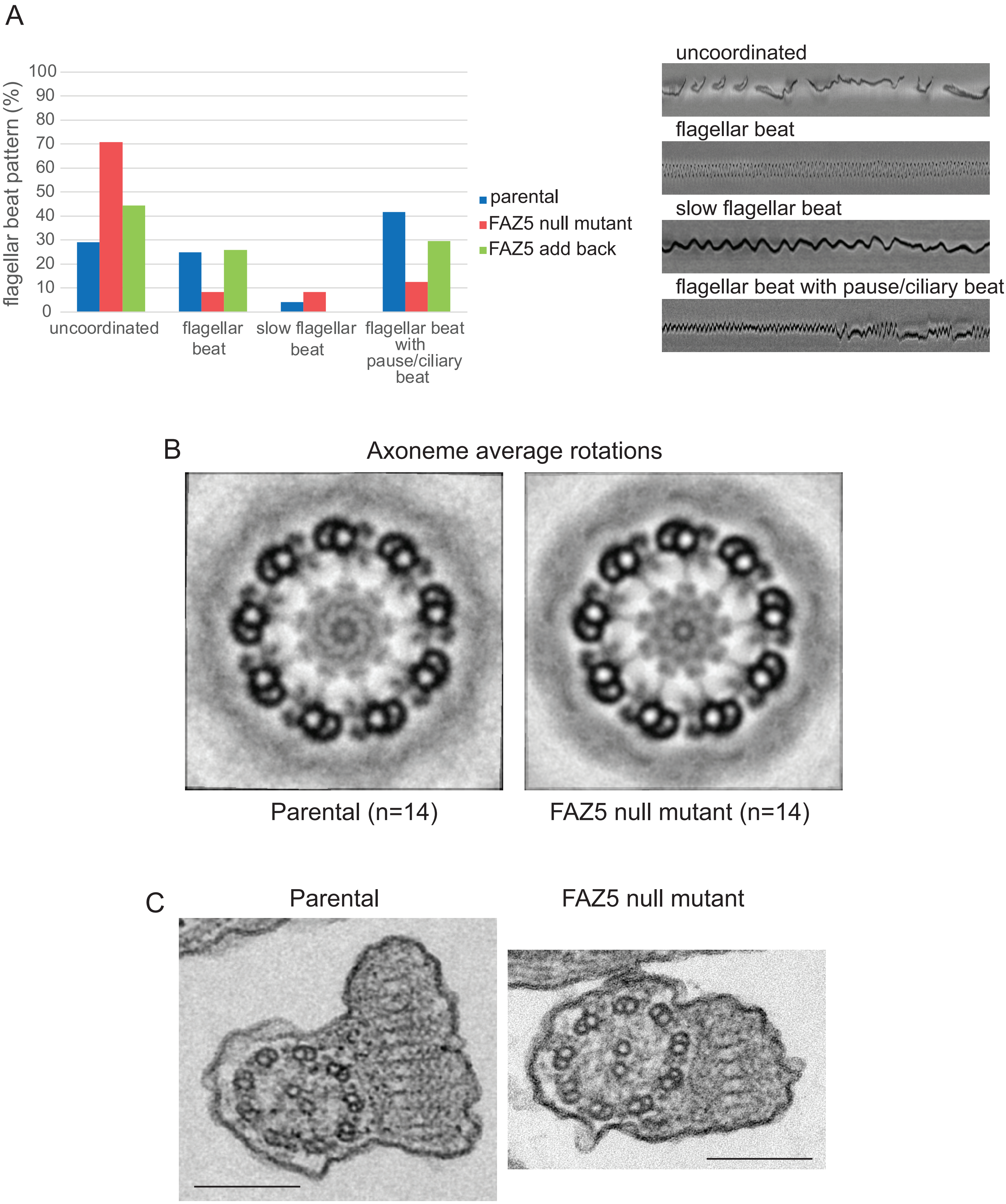

Figure S3 related to Figure 2. A) Analysis of flagellar beat patterns from high speed videos for the parental (n=24), FAZ5 null mutant (n=24) and FAZ5 (n=27) add back cells with example tracks for each category of beating pattern. B) Markham rotation of axonemal cross sections from the parental cells and the FAZ5 null mutant, showing that there is no observable difference between the axoneme structure. C) Example TEM images of axoneme and PFR cross sections, showing no observable changes in axoneme or PFR structure. Scale bar is 200 nm.

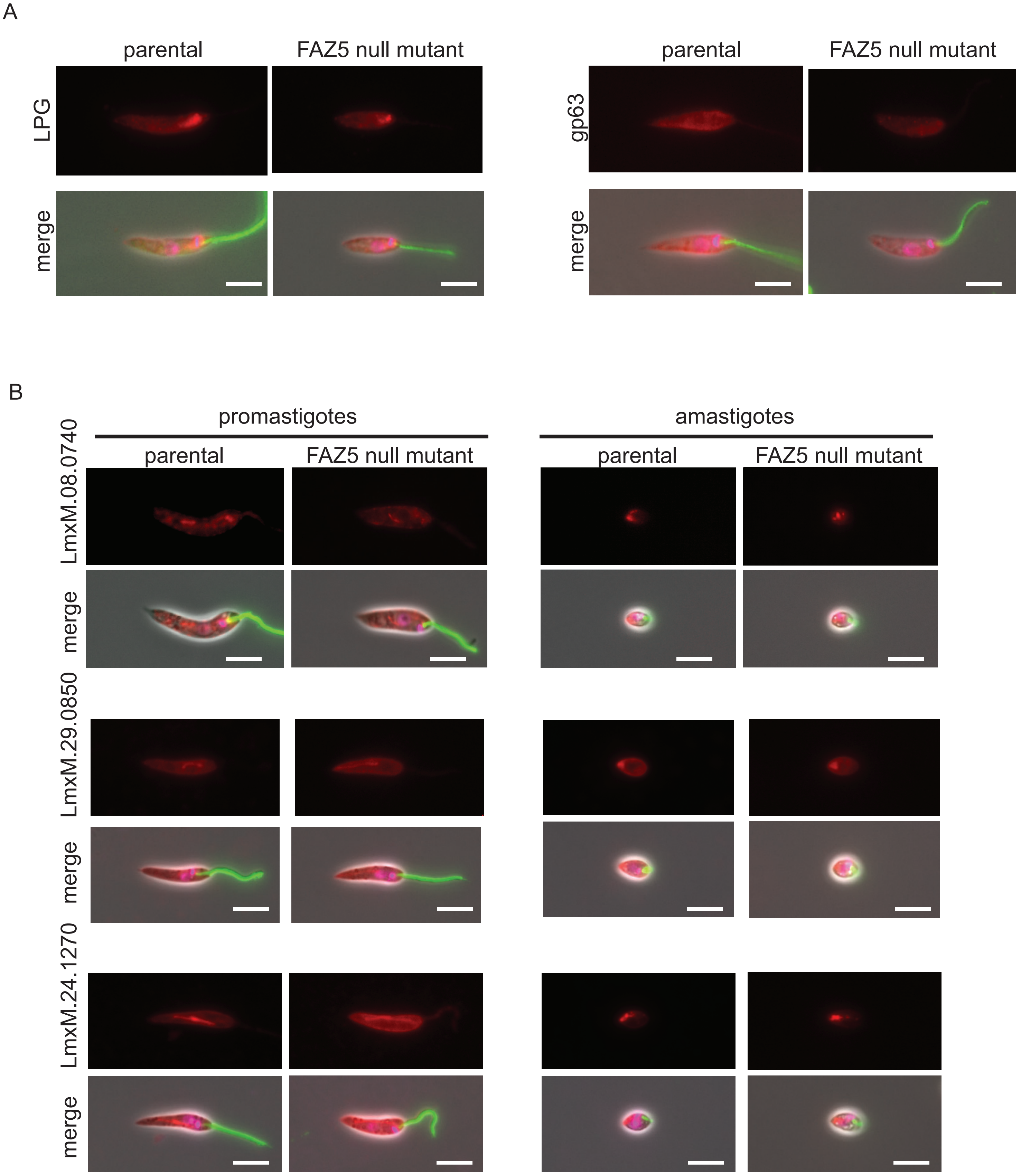

Figure S4 related to Figure 3. A) Images of parental and FAZ5 null mutants stained with LPG and gp63 antibodies (red). The flagellum membrane protein SMP1 is tagged with eGFP (green) and the DNA is stained with Hoescht 33342 (blue). Scale bar represents 5 μm. B) Images of amastins, LmxM.08.0740, LmxM.29.0850 and LmxM.24.1270, tagged with dTomFP (red) in parental and FAZ5 null mutant cells for both promastigotes and axenic amastigotes. The flagellum membrane protein SMP1 is tagged with eGFP (green) and the DNA is stained with Hoescht 33342 (blue). Scale bar represents 5 μm.

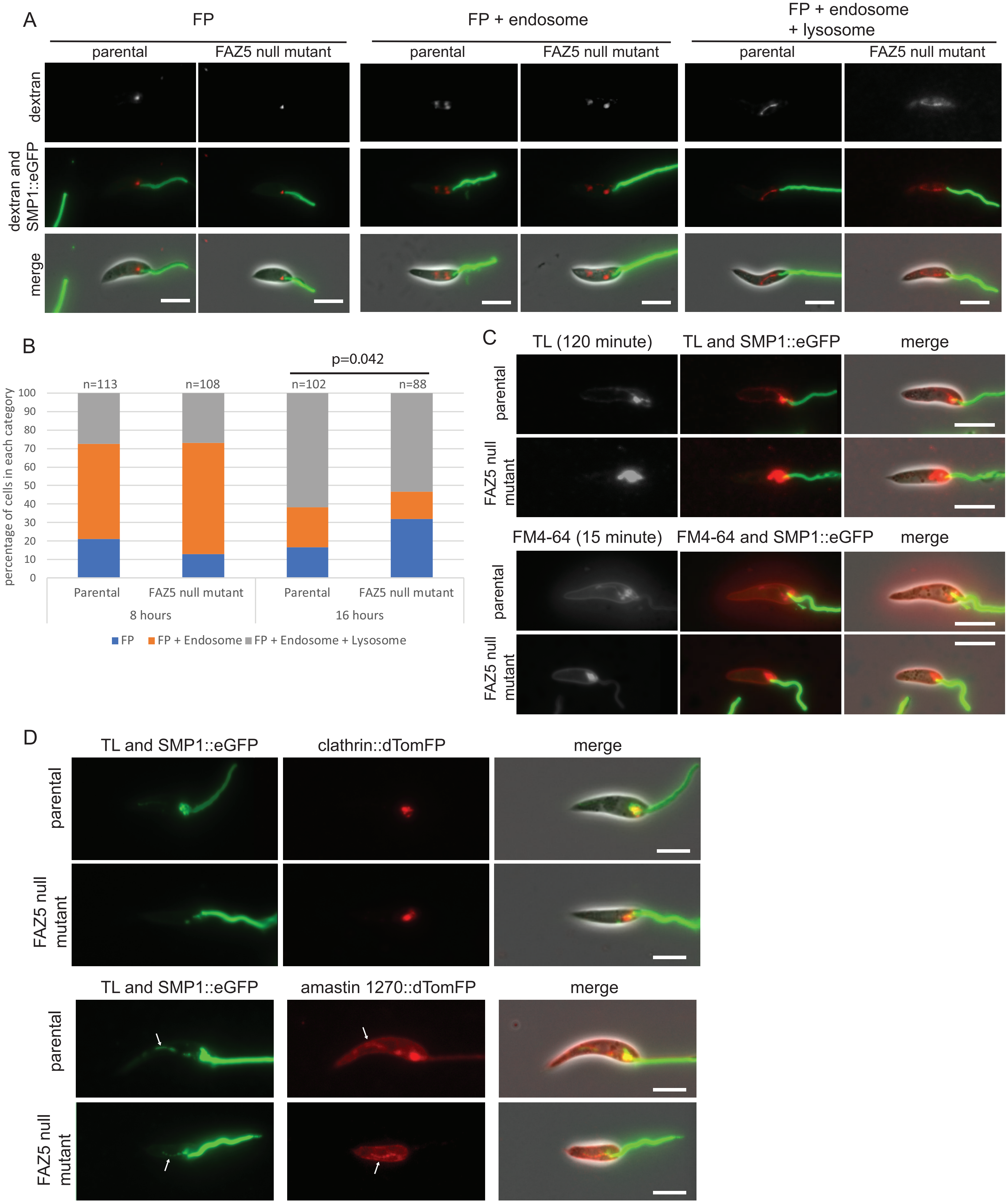

Figure S5 related to Figure 3. A) 10 kDa dextran uptake assay with parental and FAZ5 null mutant promastigotes. Promastigotes were incubated with 10 kDa dextran over a 16 hours timecourse and images captured at 8 and 16 hours. Three major categories of dextran localisation were observed - FP, FP and endosome, and FP, endosome and lysosome. The flagellum membrane protein SMP1 is tagged with eGFP (green). Scale bar represents 5 µm. B) Proportion of each category observed at each time point for both parent and FAZ5 null mutants. The assay was done independently three times and results from a representative experiment are shown. P value was calculated using a chi-squared test. C) Example micrographs of parental and FAZ5 null mutant cells from TL and FM4-64 uptake assays. At the 120 minute and 15 minute time point for TL and FM4-64 (red) respectively there is a stronger signal from the FP/endosome in the FAZ5 null mutants than the parental cells. The flagellum membrane protein SMP1 is tagged with eGFP (green). Scale bar represents 5 µm. D) Parental and FAZ5 null mutants expressing clathrin and amastin 1270 tagged with dTomFP (red) were incubated with green tomato lectin (TL - green). Overlap of TL signal with clathrin (endosome) and amastin 1270 (lysosome - white arrow) was seen. The flagellum membrane protein SMP1 is tagged with eGFP (green). Scale bar represents 5 µm.

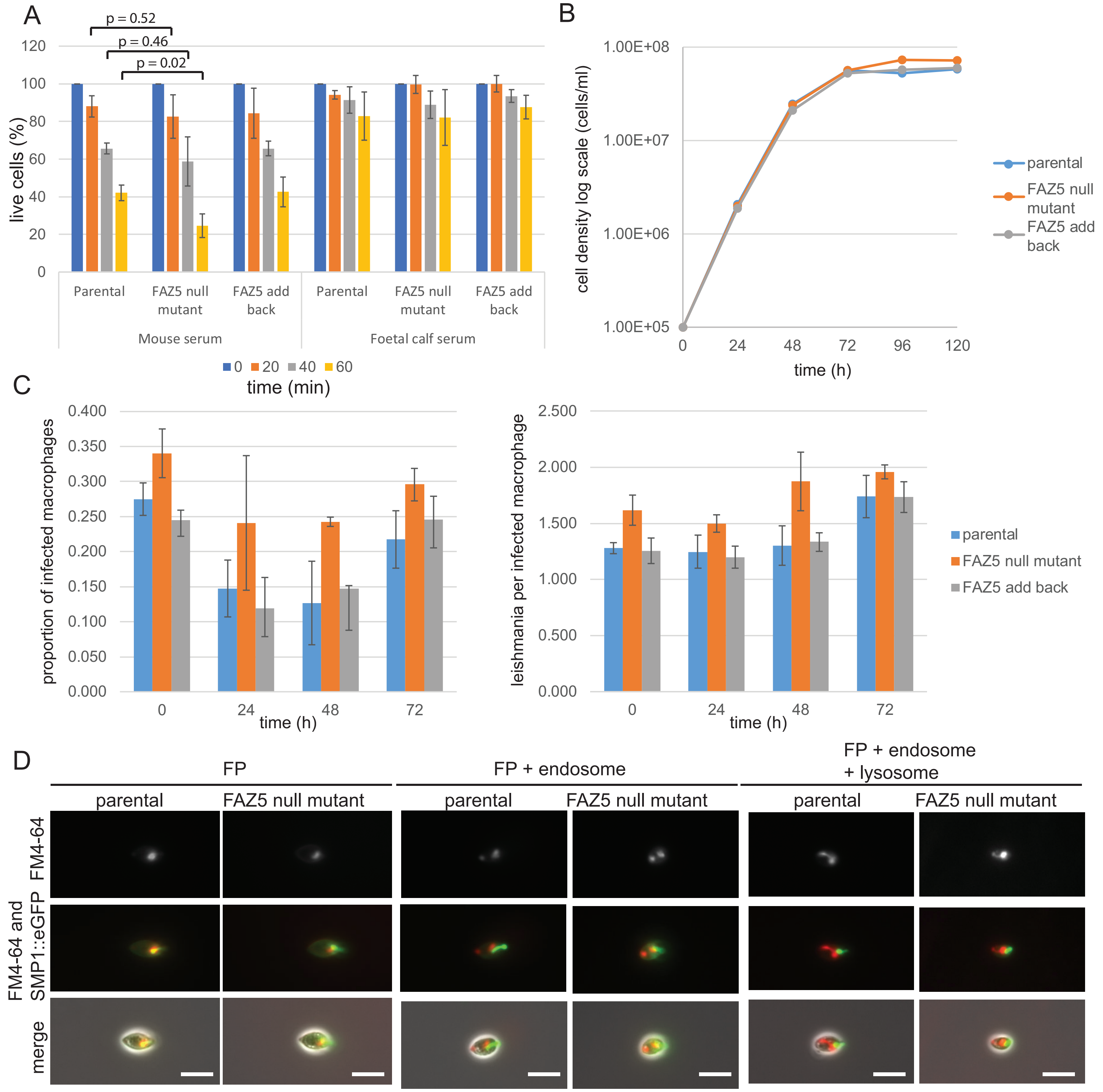

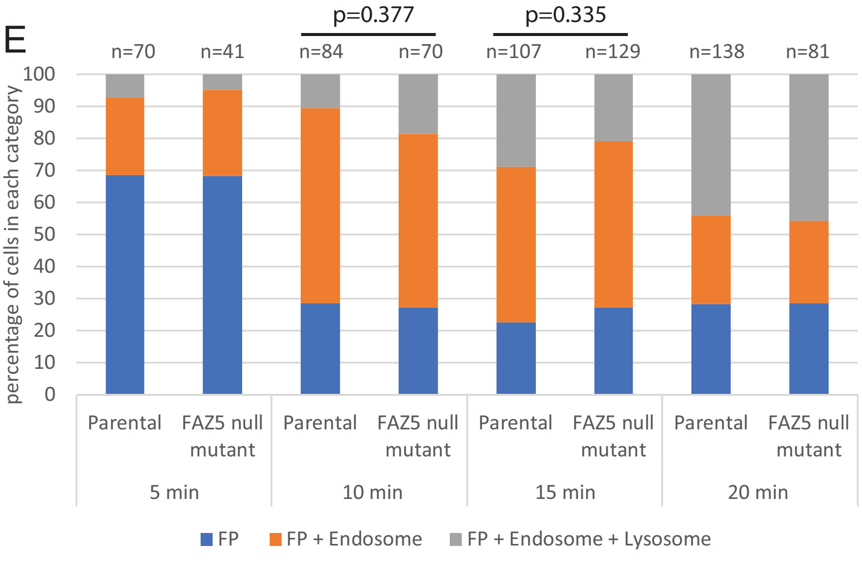

Figure S6 related to Figure 6. A) Effect of serum on promastigote cells. Parental, FAZ5 null mutant and FAZ5 add back cells were incubated with 20% mouse or foetal calf serum and killing measured by counting live cells at each time point. B) *Leishmania* macrophage infections. Growth curve of parental, FAZ5 null mutant and FAZ5 add back cells to stationary phase - average of 3 replicates. C) Proportion of infected macrophages and the number of leishmania per infected macrophage at 0, 24, 48, 72 hours post infection - 0 h time point is after 2 hours of infection and removal of cells not taken up. For each time point 497-1076 macrophages were analysed, n=3 for parental, FAZ5 add back, n=2 for FAZ5 null mutant. Error bars show standard deviation. D) FM4-64 uptake assay with parental and FAZ5 null mutant axenic amastigotes. Amastigotes were incubated with FM4-64 (red) over a 20 minute time course and images captured at 5 minute intervals. The flagellum membrane protein SMP1 is tagged with eGFP (green). Scale bar represents 5 μm. Three major categories of FM4-64 localisation were observed - FP, FP and endosome, and FP, endosome and lysosome. E) Proportion of each category observed at each time point for both parent and FAZ5 null mutants. The assay was done independently three times and results from a representative experiment are shown. P value was calculated using a chi-squared test.

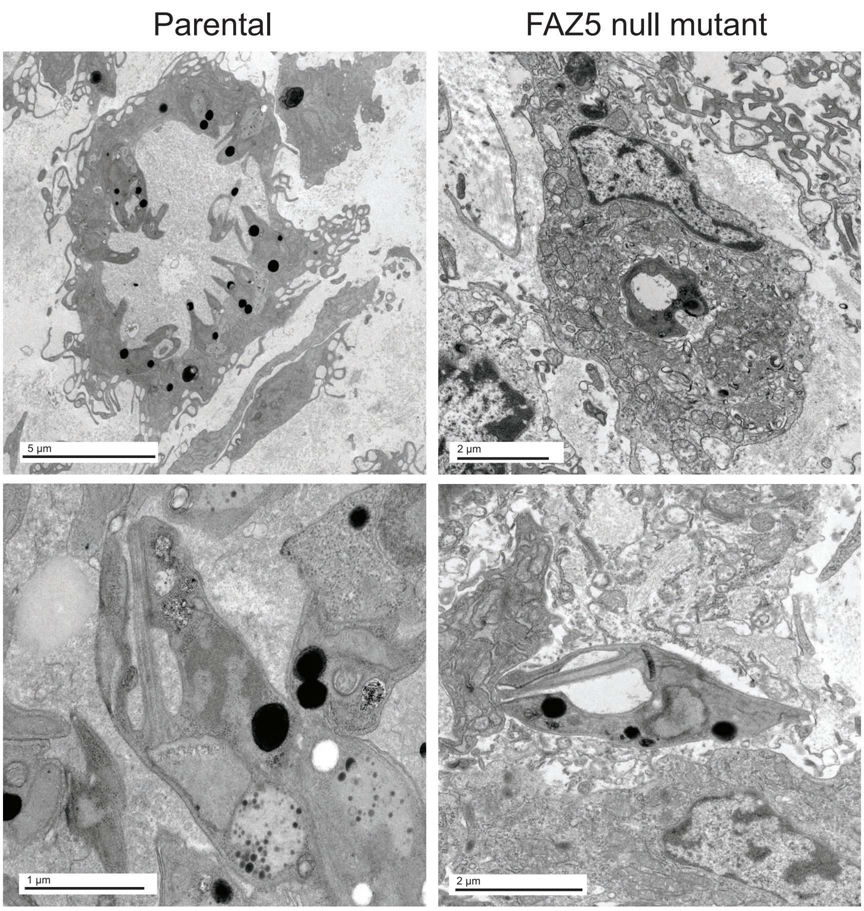

Figure S7 related to Figure 6. FAZ5 null mutant is able to differentiate and infect macrophages in the mouse footpad. TEM images of sections through mouse footpads infected with either parental cells or FAZ5 null mutant.
